# BET bromodomain inhibitors PFI-1 and JQ1 are identified in an epigenetic compound screen to enhance *C9ORF72* gene expression and shown to ameliorate *C9ORF72*-associated pathological and behavioral abnormalities in a C9ALS/FTD model

**DOI:** 10.1186/s13148-021-01039-z

**Published:** 2021-03-16

**Authors:** Esteban Quezada, Claudio Cappelli, Iván Diaz, Nur Jury, Nicholas Wightman, Robert H. Brown, Martín Montecino, Brigitte van Zundert

**Affiliations:** 1 Institute of Biomedical Sciences (ICB), Faculty of Medicine & Faculty of Life Sciences, Universidad Andres Bello, Santiago, Chile; 2 Department of Neurology, University of Massachusetts Medical School (UMMS), Worcester, MA USA; 3 FONDAP Center for Genome Regulation, Santiago, Chile; 4 CARE Biomedical Research Center, Faculty of Biological Sciences, Pontificia Universidad Católica de Chile, Santiago, Chile

**Keywords:** C9ORF72, Epigenetics, BET inhibitors, DPR, RNA foci, Memory

## Abstract

**Background:**

An intronic GGGGCC (G4C2) hexanucleotide repeat expansion (HRE) in the *C9ORF72* gene is the most common cause of amyotrophic lateral sclerosis (ALS) and frontotemporal dementia (FTD), referred to as C9ALS/FTD. No cure or effective treatment exist for C9ALS/FTD. Three major molecular mechanisms have emerged to explain C9ALS/FTD disease mechanisms: (1) C9ORF72 loss-of-function through haploinsufficiency, (2) dipeptide repeat (DPR) proteins mediated toxicity by the translation of the repeat RNAs, and more controversial, (3) RNA-mediated toxicity by bidirectional transcription of the repeats that form intranuclear RNA foci. Recent studies indicate a double-hit pathogenic mechanism in C9ALS/FTD, where reduced C9ORF72 protein levels lead to impaired clearance of toxic DPRs. Here we explored whether pharmacological compounds can revert these pathological hallmarks in vitro and cognitive impairment in a C9ALS/FTD mouse model (C9BAC). We specifically focused our study on small molecule inhibitors targeting chromatin-regulating proteins (epidrugs) with the goal of increasing *C9ORF72* gene expression and reduce toxic DPRs.

**Results:**

We generated luciferase reporter cell lines containing 10 (control) or ≥ 90 (mutant) G4C2 HRE located between exon 1a and 1b of the human *C9ORF72* gene. In a screen of 14 different epidrugs targeting bromodomains, chromodomains and histone-modifying enzymes, we found that several bromodomain and extra-terminal domain (BET) inhibitors (BETi), including PFI-1 and JQ1, increased luciferase reporter activity. Using primary cortical cultures from C9BAC mice, we further found that PFI-1 treatment increased the expression of V1-V3 transcripts of the human mutant *C9ORF72* gene, reduced poly(GP)-DPR inclusions but enhanced intranuclear RNA foci. We also tested whether JQ1, an BETi previously shown to reach the mouse brain by intraperitoneal (i.p.) injection, can revert behavioral abnormalities in C9BAC mice. Interestingly, it was found that JQ1 administration (daily i.p. administration for 7 days) rescued hippocampal-dependent cognitive deficits in C9BAC mice.

**Conclusions:**

Our findings place BET bromodomain inhibitors as a potential therapy for C9ALS/FTD by ameliorating *C9ORF72*-associated pathological and behavioral abnormalities. Our finding that PFI-1 increases accumulation of intranuclear RNA foci is in agreement with recent data in flies suggesting that nuclear RNA foci can be neuroprotective by sequestering repeat transcripts that result in toxic DPRs.

**Supplementary Information:**

The online version contains supplementary material available at 10.1186/s13148-021-01039-z.

## Background

Amyotrophic lateral sclerosis (ALS) and frontotemporal dementia (FTD) are two devastating, progressive neurodegenerative diseases with clinically distinct entities. ALS, also known as Lou Gehrig’s disease, is a motoneuron disease affecting spinal cord, brainstem and cranial motoneurons, leading to death by respiratory failure within 3–5 years of diagnosis [1]. The majority of ALS patients have the sporadic form of the disease (sALS), but about 10% of the cases have familial ALS (fALS), which is associated with pathogenic mutations in genes such as superoxide dismutase 1 (*SOD1*), transactive response DNA-binding protein 43 (*TARDBP* encoding TDP43), and *C9ORF72*, which is characterized by an intronic hexanucleotide expansion. FTD, the second most common cause of early dementia (< 65 years) after Alzheimer’s disease [2], is characterized by progressive deficits in behavior, language and executive functions due to a neuronal atrophy in the frontal and temporal cortices [1, 3, 4]. Despite these distinct clinical entities, ALS and FTD patients also can present clinical and histopathological overlaps and share similar genetic signatures, including pathogenic mutations in *TARDBP* and *C9ORF72* [1, 2, 5–7]. In fact, patients harboring mutations in *C9ORF72* can suffer from ALS, FTD or a mixture of both, explaining the wide clinical diversity of both diseases. ALS and FTD have been classified as the extreme ends of the ALS/FTD spectrum; individuals carrying mutations in *C9ORF72* are collectively referred to as C9ALS/FTD patients. Currently, there is no cure and no effective treatments to halt, or reverse, the progression C9ALS/FTD, or for non-C9ORF72 related ALS patients, partly because of an incomplete understanding of the causative pathomechanisms of these disorders.

Since the discovery that a hexanucleotide repeat expansion (HRE) composed of the GGGGCC (G_4_C_2_) sequence within the first intron of the *C9ORF72* gene is the most common cause of C9ALS/FTD [8, 9], major efforts have been undertaken to comprehend the underlying mechanism of this mutation (reviewed in [7, 10–14]). While the number of HRE is up to 25 in healthy subjects, C9ALS/FTD patients usually carry hundreds to thousands HRE in tandem in one of the alleles of the gene, forming higher-order DNA structures called G-quadruplexes [11, 12, 15, 16]*.* Three major pathogenic mechanisms have been implicated in C9ALS/FTD. First, the most widely accepted disease mechanism involves *r*epeat *a*ssociated *n*on-ATG (RAN) translation of the sense and antisense *C9ORF72* transcripts leading to the accumulation of toxic dipeptide repeat (DPR) proteins that form neuronal inclusions in the brains of C9ALS/FTD patients [17–21]. The five DPRs contain glycine (G), alanine (A), arginine (R) and/or proline (P) residues produced from the repeat transcripts. Of the five DPRs, poly(GA) and poly(GR) are uniquely produced from the sense (G_4_C_2_) transcripts, while poly(PA) and poly(PR) are uniquely generated from the antisense (G_2_C_4_) transcripts; poly(GP) can be produced from both transcripts. Toxicity of DPRs is specifically high for arginine-containing DPR proteins and involves many processes, including deficits in nucleocytoplasmic transport and RNA processing [11, 12]. Second, the sense and antisense transcripts generated by the bidirectional transcription of the expanded *C9ORF72* gene form nuclear repeat-containing RNA inclusions, or RNA foci, a hallmark in C9ALS/FTD patient tissues [8, 19, 22–25]. However, whether RNA foci are toxic [11] or neuroprotective under certain circumstances [26, 27] remains unclear. Third, C9ORF72 loss-of-function leads to decreased *C9ORF72* mRNA and protein levels in C9ALS/FTD patient tissues and derived cell lines [8, 9, 28–35]. Reduced *C9ORF72* gene expression is related to epigenetic alterations at this locus, with an enrichment of several repressive histone modifications, including trimethylation of lysine 9 and 27 of histone H3 (H3K9me3, H3K27me3) as well as DNA methylation in the *C9ORF72* gene promoter and/or HRE sequence [36–42]. Additionally, the generation of full length *C9ORF72* transcripts is impaired by DNA G-quadruplexes that result in abortive transcription [12, 15, 16]*.* Loss of C9orf72 in worm and zebrafish models induces motoneuron death and motor deficits [43, 44]. While reduction or elimination of C9ORF72 also can lead to neuronal dysfunction and death in human and mouse cultures [45], depletion of C9orf72 alone in mice does not lead to neurodegeneration or CNS behavioral changes [46–52]. These results indicate that C9ORF72 loss-of-function by itself is not sufficient to drive neurodegeneration in mice [53]. Moreover, recent studies suggest a double-hit pathogenic mechanism in C9ALS/FTD, where reduced C9ORF72 protein levels leads to autophagy deficits and hence impaired clearance of toxic DPRs [14, 54]. Together, these data indicate that increasing *C9ORF72* gene expression could subsequently reduce the presence of toxic DPRs.

Here, we addressed whether pharmacological compounds can improve or even reverse the C9ALS/FTD disease phenotypes. We specifically focused our study on small molecules inhibiting chromatin-regulating proteins (epidrugs) to increase *C9ORF72* gene expression and hence potentially ameliorate *C9ORF72*-associated pathological and behavioral abnormalities. A previous study by Zeier et al. [42] used a semi-high-throughput screen to identify siRNAs and small molecule inhibitors of epigenetic modifier proteins that increase the expression of *C9ORF72* RNA in fibroblasts, lymphocytes and induced pluripotent stem cells (iPSC)-derived motoneurons from C9ALS patients. This study provides evidence that bromodomain and extra-terminal domain (BET) inhibitors (BETi), including JQ1 and I-BET151 (PFI-1 was not tested), as regulators of the *C9ORF72* locus in C9ALS. BET proteins (BRD2, BRD3, BRD4 and BRDT) play a key role in transcription and chromatin remodeling (see discussion). In our study, we tested 14 selective small molecules that target and inhibit the activity of bromodomains as well as chromodomains and histone-modifying enzymes. To evaluate the effect of these epidrugs we took advantage of novel SH-SY5Y luciferase reporter cell lines comprising 10 (control) or ≥ 90 (mutant) G4C2 HRE. It was found that the bromodomain and BETi PFI-1 and JQ1 were the most effective epidrugs in enhancing the activity of the mutant human *C9ORF72* gene. We also examined primary cortical neurons from a C9ALS/FTD transgenic mouse model, C9BAC, that carries a bacterial artificial chromosome (BAC) containing a fragment of the human *C9ORF72* gene derived from a familial ALS/FTD patient [55]. Treatment of these cells with PFI-1 enhanced expression of the human mutant *C9ORF72* gene, increased accumulation of nuclear RNA foci and reduced poly(GP)-DPR inclusions. Finally, treatment with JQ1 (daily i.p. administration for 7 days) reduced hippocampal-dependent cognitive impairments in C9BAC mice. Together, our data demonstrate that BETi can increase *C9ORF72* gene expression and reduce the presence of toxic DPRs in vitro. In vivo, these compounds can reduce memory deficits in a C9ALS/FTD mouse model.

## Results

### Bromodomain inhibitors enhance transcription of the human *C9ORF72* gene stably integrated in SH-SY5Y cells

**Fig. 1:**
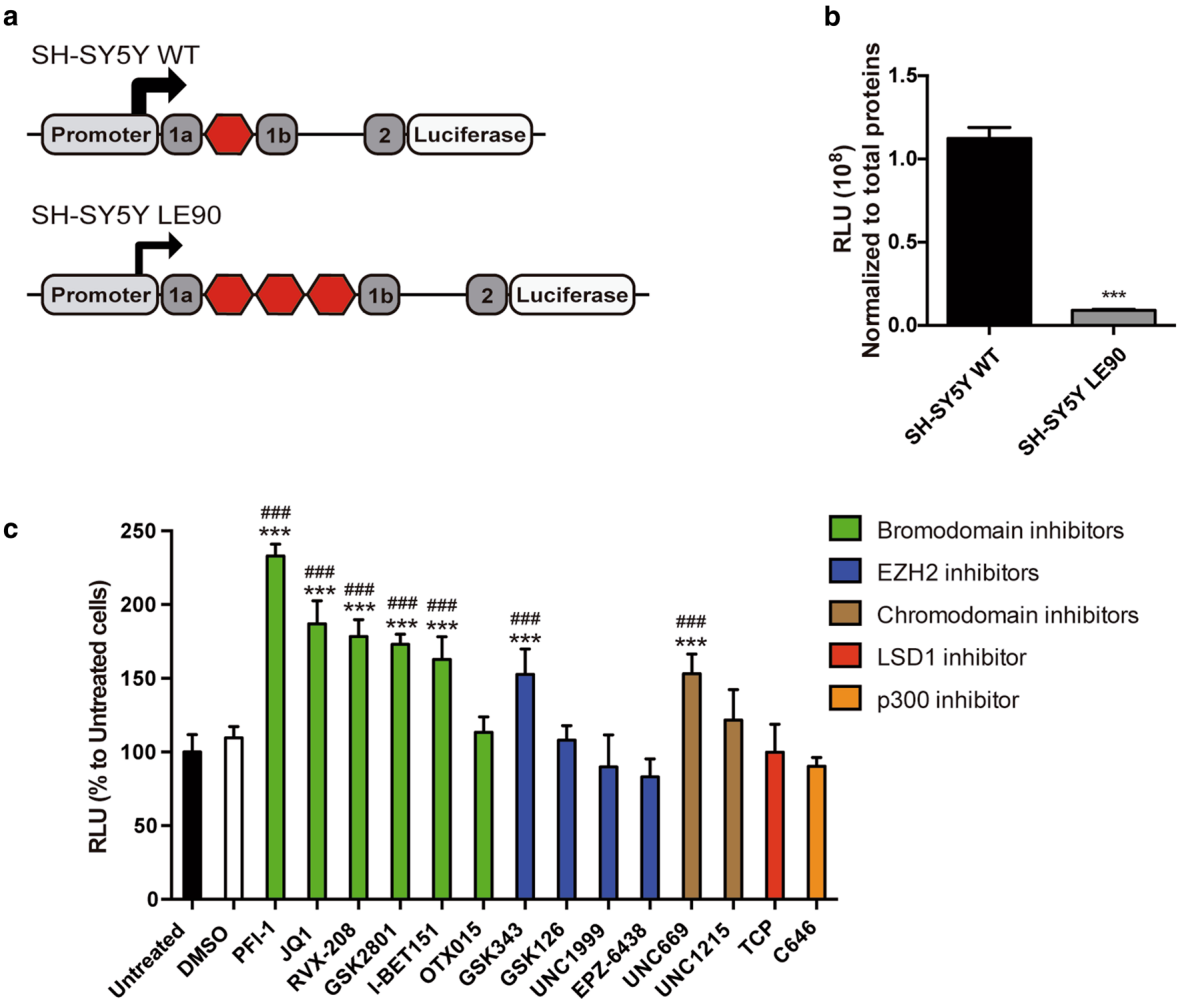
Bromodomain inhibitors enhance *C9ORFF72* gene promoter-driven expression in the SH-SY5Y LE90 G4C2 reporter cell line. **a** Schematic representation of the two SH-SY5Y cell lines stably expressing a segment of the human *C9ORF72* gene, including the promoter region and exons 1a through 2, driving the expression of a luciferase reporter gene. Between exons 1a and 1b, increasing G4C2 hexanucleotide repeat expansions (red hexagons) were inserted: 10 (SH-SY5Y WT) or 90 (SH-SY5Y LE90). **b** Quantification of the basal luciferase activity in each cell line. Values were normalized to total protein concentration and displayed as relative luciferase unit (RLU). Bars represent mean ± S.E.M. ***P < 0.001 relative to SH-SY5Y WT cells, unpaired Student’s *t*-test (*n* = 3 independent experiments). **c** Quantification of luciferase activity at the SH-SY5Y LE90 cells following an incubation for 24 h with 14 selective small molecule inhibitors targeting bromodomains (green bars), histone methyltransferase EZH2 (blue bars), chromodomains (brown bars), the histone demethylase LSD1 (red bar) and the histone acetyltransferase p300 (orange bar). As a control, SH-SY5Y LE90 cells were also treated with 0.05% (v/v) DMSO (white bar), the same concentration used as the vehicle for most of the epidrugs. Luciferase activity in the treated SH-SY5Y LE90 cells was normalized to untreated SH-SY5Y LE90 cells (black bar). Bars represent mean ± S.E.M. ***P < 0.001 relative to DMSO, or ^###^*P* < 0.001 relative to Untreated, one-way ANOVA (*n* = 3 independent experiments)

To assess the effect of targeting different chromatin-regulators on the expression of the human *C9ORF72* gene, we first generated an in vitro model of C9ALS/FTD that facilitates drug screenings. Specifically, we stably transfected SH-SY5Y cells with a construct that contains part of the human *C9ORF72* gene, including the promoter region (4 kb) and exons 1a through 2, cloned upstream of the luciferase reporter gene (Fig. 1a). Between exons 1a and 1b, these constructs contain two different sizes of G4C2 repeat motifs: a control construct including 10 repeats (SH-SY5Y WT) and a pathological version containing 90 repeats (SH-SY5Y LE90) (Fig. 1a). As expected, the basal luciferase activity of SH-SY5Y LE90 cells was significantly reduced compared to the control line SH-SY5Y WT (Fig. 1b). Our screening assay included 14 selective small molecules that target and inhibit the activity of bromodomains, chromodomains and histone-modifying enzymes, including the histone methyltransferase EZH2, the histone demethylase LSD-1 and the histone acetyltransferase p300 (Fig. 1c). These epidrugs were selected because they target proteins that belong to principal epigenetic regulatory pathways that control gene expression in a large number of biological systems under multiple physiological cues [56–58].

Details on these epidrugs, including their molecular structure, target proteins, and final concentrations used to treat the SH-SY5Y cells for 24 h are shown in Additional file 1: Table S1. Dose response curves (ranging from 1 nM to 35 µM) were generated for each compound (always freshly prepared). Optimal results for most of these epidrugs were reached when applied for 24 h at a concentration of 5 µM. Exceptions were found for RXV208 (25 µM), OTX015 (2.5 µM) and GSK343 (0.5 µM). At these selected concentrations, these compounds induced the highest luciferase activity without causing any detectable cytotoxicity (not shown). As necessary controls, SH-SY5Y cells were either untreated or treated with vehicle DMSO. The DMSO volume concentrations (v/v) ranged from 0.005% to 0.25% (see Methods) and did not induce cytotoxicity or changes in luciferase activity compared to untreated SH-SY5Y cells (Fig. 1c for 0.5% DMSO and data not shown).

All the bromodomain-related inhibitors PFI-1, (+)-JQ1 (henceforth, JQ1), RVX-208, GSK2801 and I-BET151 enhanced the luciferase activity in SH-SY5Y LE90 cells, compared to the controls (Fig. 1c). PFI-1, JQ1, RVX-208 and I-BET151 are selective and potent BETi, whereas GSK2801 is a selective inhibitor of the bromodomain adjacent zinc finger (BAZ2) [59, 60]. Some bromodomain and BET inhibitors were also tested in a second disease model cell line containing 200 G4C2 repeats between exons 1a and 1b of the human *C9ORF72* gene (SH-SY5Y LE200); the proliferation rates of SH-SY5Y LE200 cells were significantly lower compared to the SH-SY5Y LE90 cells and therefore used for selected epidrugs. As expected, SH-SY5Y LE200 cells also exhibited a strongly reduced basal luciferase activity compared to the control line SH-SY5Y WT (Additional file 1: Fig. S1A, B). In line with the results found in SH-SY5Y LE90 cells, we found that bromodomain inhibitors PFI-1, RVX-208, I-BET151 significantly enhanced the luciferase activity in SH-SY5Y LE200 (Additional file 1: Fig. S1C). We also found that OTX015 (another BETi) did significant increase luciferase activity in SH-SY5Y LE200 cells, while this epidrug was not effective in the SH-SY5Y LE90 cells (Fig. 1c; Additional file 1: Fig. S1C). Conversely, GSK2801 was able to increase luciferase activity in the SH-SY5Y LE90 cells, but not in the SH-SY5Y LE200 cells.

Using the SH-SY5Y LE90 cells, we also tested several inhibitors for chromodomains and the histone-modifying enzymes EZH2, LSD-1 and p300. Only the chromodomain inhibitor UNC669 and the EZH2 inhibitor GSK343 significantly increased luciferase activity, while other epidrugs targeting similar domains/enzymes had no effect (Fig. 1c). Based on these results, we concluded that the BETi PFI-1 and JQ1 are the most potent chemical probes mediating increased transcription of the human *C9ORF72* gene in the SH-SY5Y luciferase reporter cell lines.

### PFI-1 mediates an increased expression of three transcript variants of the human mutant *C9ORF72* gene in C9BAC primary cortical cultures

**Fig. 2:**
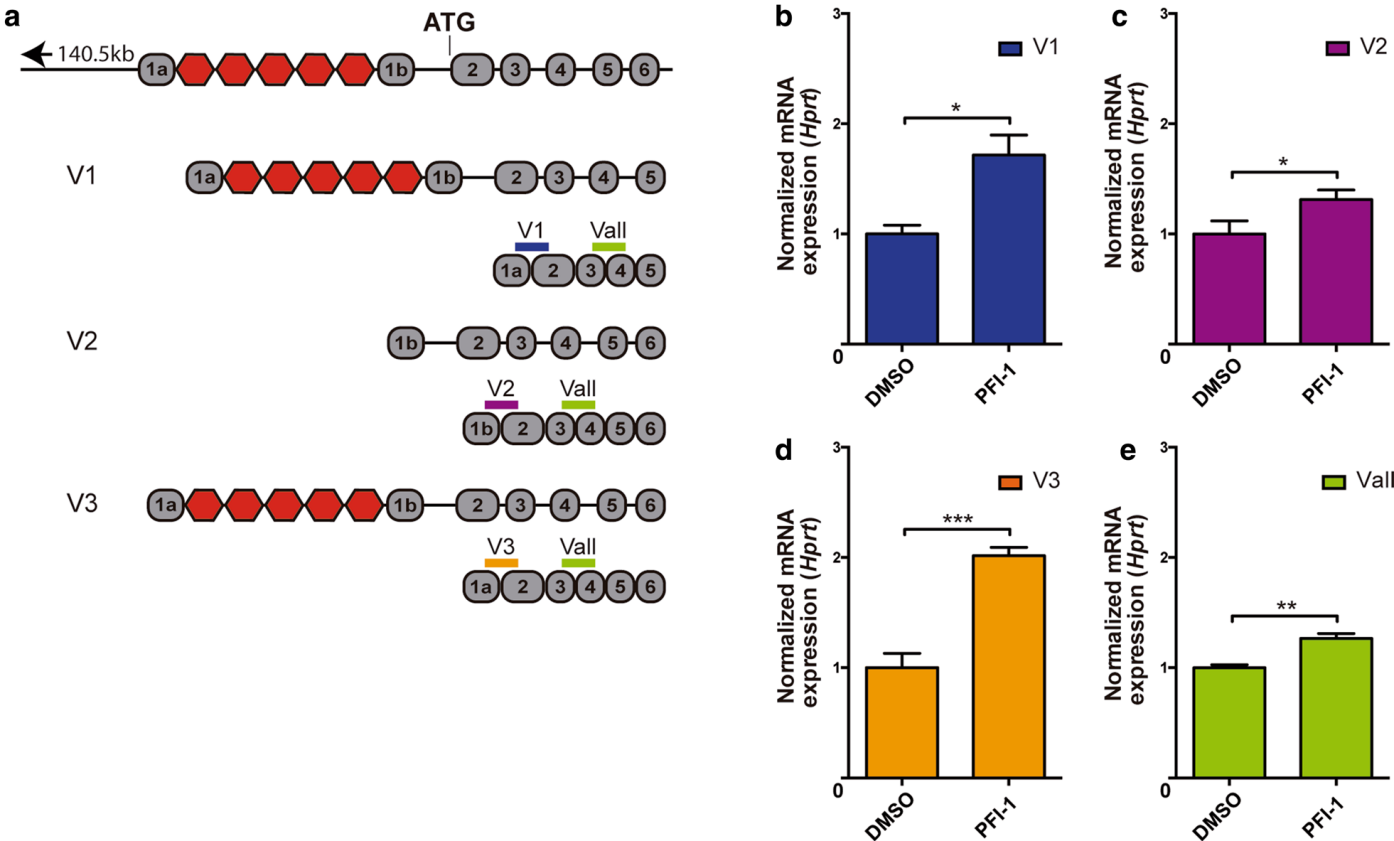
The bromodomain inhibitor PFI-1 enhances the expression of the *C9ORF72* transcript variants. **a** Schematic representation of the BAC construct (153.2 kb) containing the human *C9ORF72* gene promoter region (140.5 kb), exons 1 to 6 and a ~ 500 G4C2 hexanucleotide repeat expansions within intron 1 (red hexagons). The regions where each TaqMan probe hybridizes to detect the transcript variants V1 (blue), V2 (purple), V3 (orange) and Vall (green) are indicated. **b**–**e** TaqMan-based expression analyses of the V1 (**b**), V2 (**c**), V3 (**d**) and Vall (**e**) *C9ORF72* transcripts. Samples were obtained from 10 DIV C9BAC primary cortical cultures treated with PFI-1 (5 µM in 0.05% DMSO) or vehicle DMSO (0.05%) for 24 h. Expression of each *C9ORF72* transcript was normalized to DMSO treatments, and internally normalized to the expression of house-keeping gene *Hprt*. In all graphs, bars represent mean ± S.E.M. *P < 0.05, **P < 0.01 and ***P < 0.001 relative DMSO treated cells, unpaired Student’s *t*-test (*n* = 3 independent experiments)

**Fig. 3:**
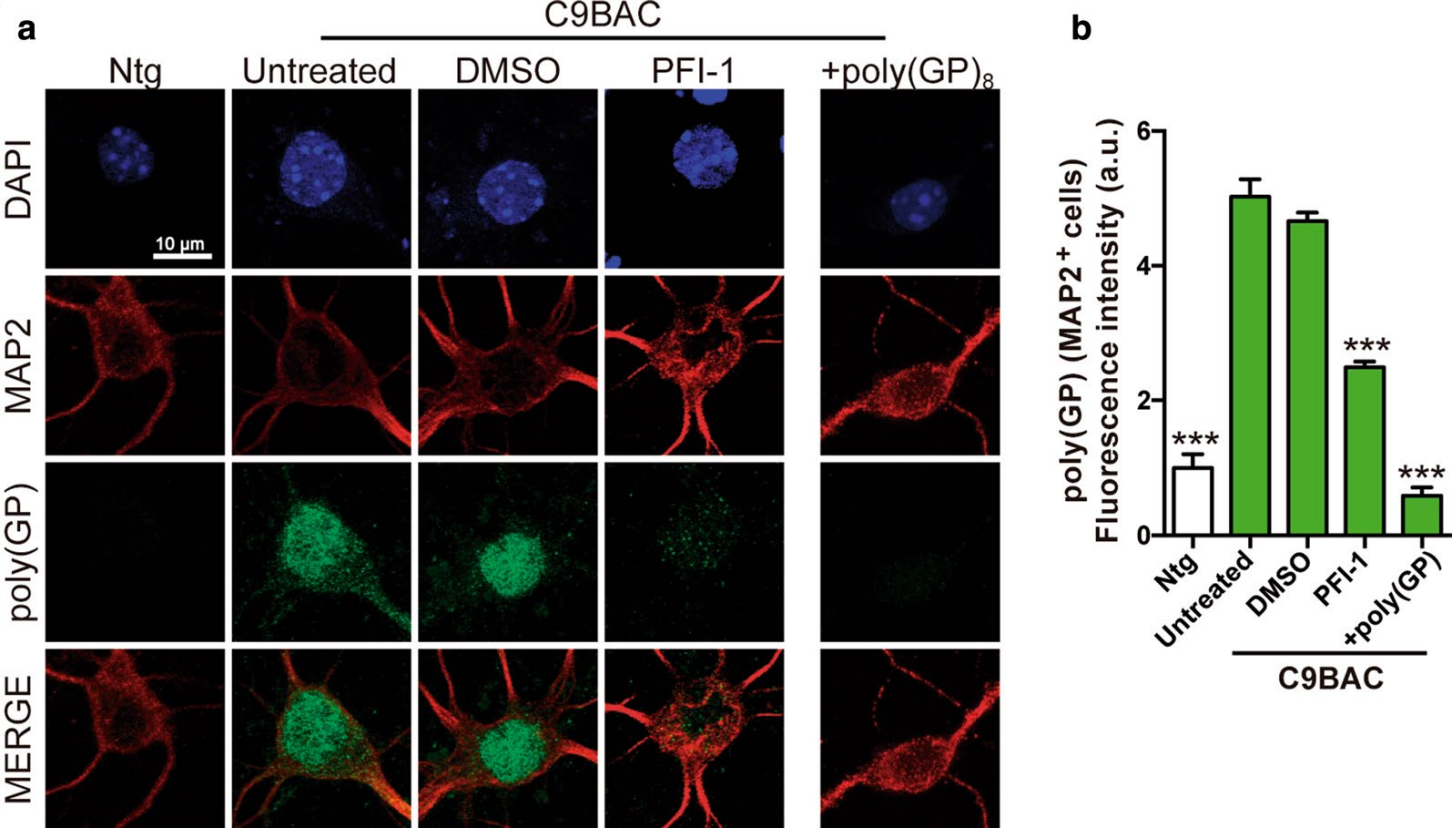
PFI-1 treatments reduce poly(GP) levels in C9BAC primary cortical neurons. **a** Representative confocal images of fixed 10 DIV cortical neurons derived from C9BAC (C9BAC) and non-transgenic (Ntg) mice analyzed by immunofluorescence labeling with an antibody against MAP2 (red) to identify neurons and an antibody against poly(GP) (green) to identify poly(GP) DPRs. DAPI (blue) staining identified the nuclei. Images show abundant presence of poly(GP) DPRs in C9BAC, but not in Ntg, cortical neurons. Poly(GP) DPRs are strongly reduced after treatment with PFI-1 (5 µM in 0.05% DMSO). Pre-incubation of the anti-poly(GP) antibody with a poly(GP) peptide led to a significant loss of the Poly(GP)-DPR signal. **b** Quantification of the nuclear fluorescence intensity at each indicated condition, normalized to the intensity in Ntg cells. Bars represent mean ± S.E.M. ***P < 0.001 relative to untreated C9BAC cells, one-way ANOVA. (*n* = 3 independent experiments with a minimum of 30 nuclei quantified for each condition)

**Fig. 4:**
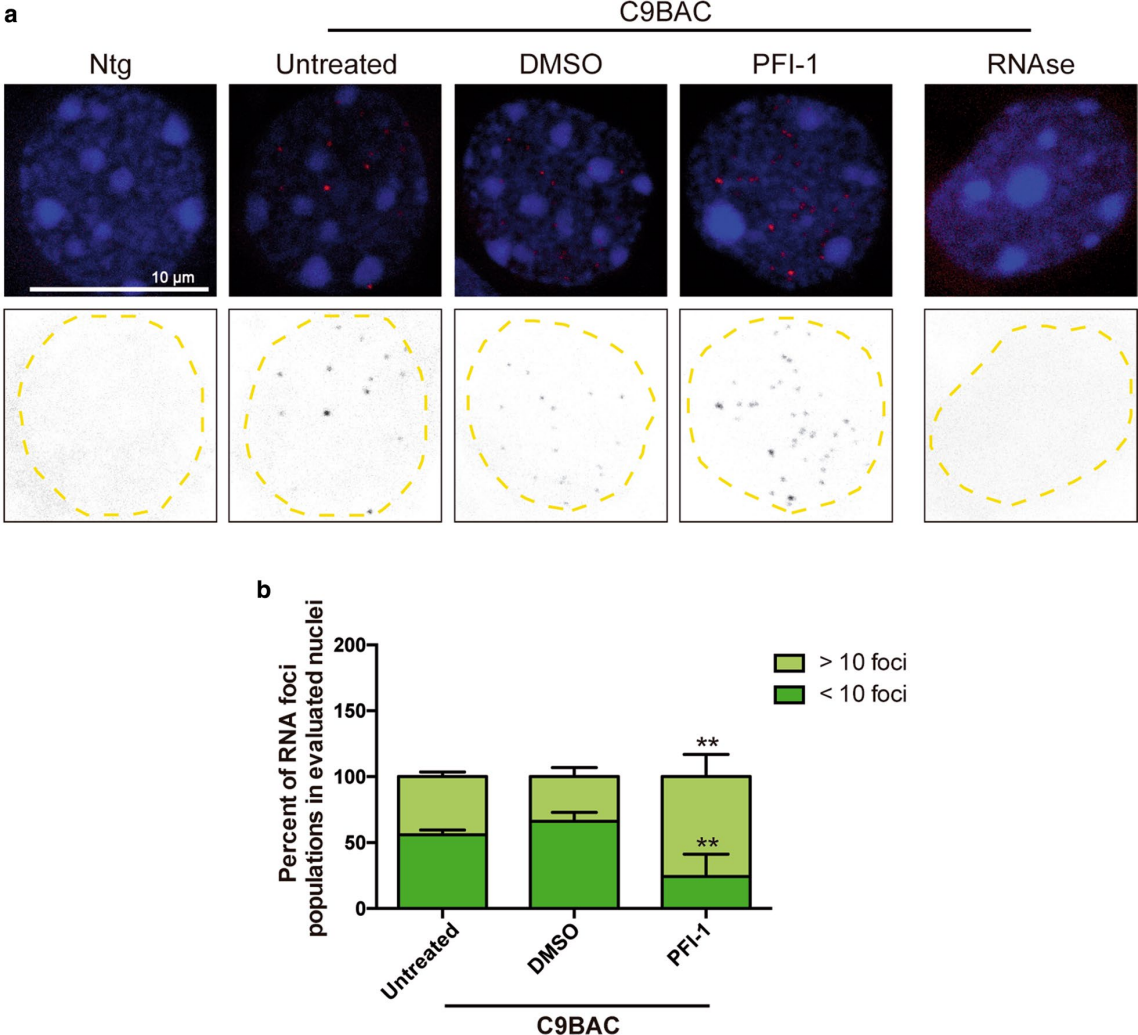
The bromodomain inhibitor PFI-1 increases sense RNA foci in C9BAC primary cortical neurons. **a** Representative confocal images (upper row) of fixed 10 DIV cortical neurons derived from C9BAC (C9BAC) and non-transgenic (Ntg) mice analyzed by RNA FISH using a 5′Cy3-tagged G2C4 DNA probe (red) targeting the sense *C9ORF72* repeat transcripts. Samples were also stained with DAPI (blue) to visualize the nucleus (blue). Black-and-white inverted images (lower row) are depicted to optimize visualization of the RNA foci. Brightfield microscopy was used to identify neurons from glial cells (not shown). Images show the presence of sense RNA foci in C9BAC (but not in Ntg) cortical neurons, that are increased in number after treatment with PFI-1 (5 µM in 0.05% DMSO) for 24 h. No RNA foci are detected after RNAse treatment. **b** Quantification of RNA foci in the different conditions as indicated. Evaluated nuclei were separated in populations of > 10 or ≤ 10 sense RNA foci. Bars show the mean percentage ± S.E.M. of each population with respect to total quantified nuclei in each condition. ***P < 0.001 relative to untreated C9BAC cells, one-way ANOVA (*n* = 3 independent experiments with a minimum of 30 nuclei quantified for each condition)

To further evaluate the effects of the BETi PFI-1 in a more complex and physiologically relevant ex vivo model of *C9ORF72*, we analyzed primary cortical cultures derived from a C9ALS/FTD mouse model. We used the transgenic C9BAC mouse model carrying a BAC that contains a segment of a human *C9ORF72* gene derived from a familial ALS/FTD patient [55]. As depicted schematically in Fig. 2a, this construct (153.2 kb) contains the human *C9ORF72* upstream and gene promoter regions (140.5 kb), exons 1 to 6 and a ~ 500 G4C2 hexanucleotide repeat expansions within intron 1. In this C9BAC mice, the truncated human *C9ORF72* gene can generate the three transcript variants V1, V2 and V3 (as well as Vall) in all tissues examined, including the cortex [55]. Primary cortical cells derived from neonatal C9BAC mice were grown in culture for 9 days in vitro (DIV) and then treated for 24 h with either 0.05% DMSO alone (Control) or with 5 μM PFI-1 in 0.05% DMSO (PFI-1). At 10 DIV, these cortical cultures were processed to evaluate the presence of *C9ORF72* transcripts (Fig. 2), poly(GP)-DPRs (Fig. 3) and RNA foci (Fig. 4). Of note, neither treatment with PFI-1 nor with DMSO caused any detectable neuronal toxicity (see for example images Fig. 3).

Detection of the human *C9ORF72* transcript variants V1, V2 and V3, as well as all variants (Vall) in the C9BAC cultures, was performed by TaqMan qRT-PCR using human-specific probes. Treatment of C9BAC neurons with PFI-1 significantly increased levels of human *C9ORF72* V1, V2, V3 and Vall mRNAs, compared to DMSO alone (Fig. 2b–d). This finding is in agreement with our results in the *C9ORF72* SH-SY5Y LE90 and SH-SY5Y LE200 cell lines (Fig. 1c; Additional file 1: Fig. S1C) and show that the BETi PFI-1 enhances the expression of the human mutant *C9ORF72* gene in C9BAC neurons.

### PFI-1 treatment reduces poly(GP)-DPR inclusions in C9BAC primary cortical neurons

Accumulation of DPRs represents a key pathological hallmark of *C9ORF72* expansion carriers. Sense and antisense *C9ORF72* transcripts are translated into five different DPR proteins by RAN translation [17] and form neuronal inclusions in the brains of C9ALS/FTD patients [18–21]. Among the five DPRs produced from the sense (G4C2) and antisense (G2C4) transcripts, the RAN translation product poly(GP) is readily detected throughout the brain of adult C9BAC mice as well as in 10 DIV primary cortical neurons derived from these mice at neonatal stages [55]. Accordingly, double immunofluorescence staining with a well-characterized anti-poly(GP) antibody [18, 55] and an anti-MAP2 antibody revealed robust poly(GP)-DPR protein inclusions in 10 DIV primary cortical neurons derived from C9BAC mice, but not from non-transgenic wild-type mice littermates (termed Ntg) (Fig. 3a). These poly(GP) inclusions were detected most frequently in the nucleus (indicated by co-localization with DAPI staining), although some cytoplasmic inclusions were also observed. To confirm the specificity of the anti-poly(GP) antibody in our cultures, the antibody was pre-incubated with an excess of poly(GP) peptide before performing the immunostaining assays in the C9BAC neurons. This pre-absorption step prevented the detection of the endogenous poly(GP) signal in C9BAC neurons, thereby confirming the specificity of our anti-poly(GP) antibody-dependent detection of poly(GP) in our in vitro C9ORF72 model (Fig. 3a, right column). We next evaluated whether the PFI-1 treatment reduced the poly(GP) inclusions in C9BAC neurons. As shown by representative confocal images (Fig. 3a) and signal quantifications (Fig. 3b), PFI-1 treatment led to a significant reduction in the intensity of the poly(GP) nuclear signals in C9BAC cortical neurons. The poly(GP) nuclear signals were found unchanged in neurons treated with the vehicle DMSO (Fig. 3a, b). Together, our in vitro data demonstrate that a treatment of C9BAC cortical neurons with BETi PFI-1 reduces poly(GP)-DPR inclusions.

### PFI-1 treatment increases intranuclear RNA foci in C9BAC primary cortical neurons

The accumulation of repeat transcripts in intranuclear RNA inclusions is another key molecular hallmark of *C9ORF72* expansion carriers; however, whether RNA foci are toxic [11] or neuroprotective under certain circumstances is unclear [26, 27]. Accumulation of both sense (G4C2) and antisense (C2G4) repeat transcripts at intranuclear RNA foci represents a hallmark of *C9ORF72* expansion carriers [19, 22–25]. C9BAC mice recapitulates this pattern showing intranuclear sense (and to lesser extent antisense) RNA inclusions throughout the mature brain (10 and 24-month-old). Thus, one to five sense foci are detected by RNA fluorescence in situ hybridization (FISH) in the majority of foci-positive nuclei of the cortical internal pyramidal layer (layer V) [55]. RNA FISH coupled with cell-type-specific immunostaining has further revealed G4C2 sense RNA foci in C9BAC neurons as well as in astrocytes [55]. To investigate whether PFI-1 treatment can reduce RNA foci in cultured C9BAC neurons, we first determined that this hallmark can be also detected in 10 DIV primary cortical cultures derived from neonatal C9BAC mice. For this, we used FISH with a 5′Cy3-tagged G2C4 DNA probe against the sense repeat expansion of *C9ORF72* transcripts, together with DAPI staining to visualize the nucleus (Fig. 4a, upper images). To optimize FISH visualization, confocal images are also shown as black-and-white inverted images (Fig. 4a, lower images). To concentrate our analysis in the FISH signal coming from the neurons, avoiding glial cells, confocal microscopy was combined with brightfield microscopy to further expose the structure of cells (not shown). As shown in representative confocal images, untreated primary cortical neurons derived from C9BAC mice exhibited sense RNA foci in their nuclei (Fig. 4a). These RNA foci signals were detected in the vast majority (> 95%) of the C9BAC neurons (data not shown), were lost following RNAse treatment and were absent in neurons obtained from non-transgenic littermates (Fig. 4a). Quantification of the number of these sense RNA foci in untreated C9BAC neurons revealed that 50% contained one to ten detectable foci (≤ 10), while the other 50% contained more than ten detectable foci (> 10) (Fig. 4b). Treatment of the cultures with DMSO did not significantly alter this proportion (Fig. 4a, b). By contrast, confocal image analysis (Fig. 4a) and subsequent quantification (Fig. 4b) revealed that treatment of the C9BAC cultures with PFI-1 led to an increase in the number of intranuclear foci in C9BAC neurons. PFI-1 induced a significant increase in the numbers of nuclei containing > 10 foci, paralleled by a significant reduction in the number of nuclei containing ≤ 10 foci. Together these results indicate that treatment of C9BAC cortical neurons with BETi PFI-1 leads to an enrichment of intranuclear RNA foci.

### JQ1 treatment reverts spatial memory impairment of C9BAC mice

**Fig. 5:**
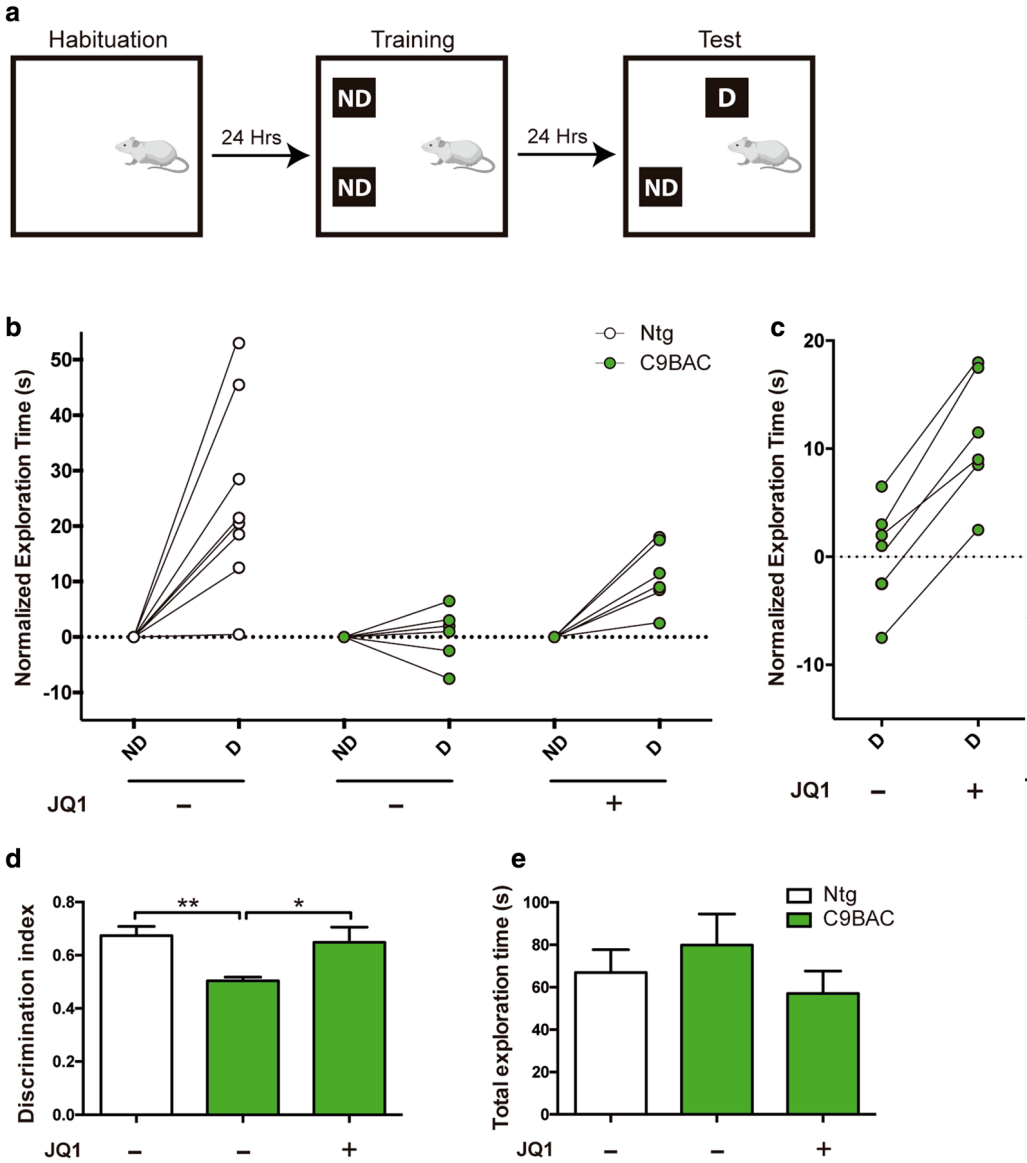
Treatment with JQ1 reverses spatial memory impairment in 6-month-old C9BAC mice. **a** Schematic representation of the object location memory (OLM) task, in which mice were subjected to habituation, followed by exposure to two similar objects (“Training”). Twenty-four h later (“Test”) the animals were evaluated by re-exposure for 10 min to the same testing area with one non-displaced object (ND) and one displaced object (D). **b** Quantification shows normalized exploration time in seconds of individual 6-month-old male C9BAC and Ntg mice, before and after JQ1 treatment (i.p. 0.5 mg/kg/day for 7 days), with non-displaced (ND) *versus* displaced (D) objects. **c** Graph shows normalized exploration time in seconds (s) of C9BAC mice before and after JQ1 treatment. **d** Discrimination index shows the time spent in exploring the displaced object (D) over the total time exploring both objects (N + D). An index of > 0.5 indicates that mice can positively discriminate between the D and ND object. The data shows that treatment of C9BAC mice with JQ1 for 7 days rescues their OLM impairment, reaching spatial memory levels comparable to Ntg mice. **e** Graph shows total exploration time and indicates that C9BAC and Ntg mice have the same exploration capacity and that JQ1 treatment does not alter this. Bars represent means ± SEM. Statistical analysis was performed by one-way ANOVA. *P < 0.05 and **P < 0.01 relative to untreated C9BAC

Previous reports demonstrate that the C9BAC mouse model does not exhibit the robust behavioral defects that are characteristic of ALS (i.e. motor deficits) or FTD (i.e. social interaction) [55]. Nevertheless, 6-month-old C9BAC mice show mild hippocampal-dependent spatial memory deficits, as measured by the object location memory (OLM) task [61]. In this simple single-trial behavioral memory task [62, 63], which relies on the rodent’s innate preference for novelty and require the hippocampus, animals are presented with a non-displaced (ND) *versus* displaced (D) object 24 h after training, while free exploring is allowed (avoiding stress) (Fig. 5a). In agreement with previous studies [61], it was found that 6-month-old C9BAC mice performed poorly on the OLM test, displaying limited exploration of the displaced object relative to control mice (Fig. 5b–d). To evaluate the effect of BETi in these animals we used JQ1, which exhibits comparable ability to stimulate the human *C9ORF72* gene expression than that shown by PFI-1 (Fig. 1). Previous studies performed a pharmacokinetic analysis of JQ1 in plasma, testis and brain following a 7-day treatment (i.p. 0.5 mg/kg/day for 7 days) in adult mice; it was determined that JQ1 is well tolerated, efficiently crosses the blood–brain barrier and remains bioavailable in brain tissue [64, 65]. Here, using the same application type, dose and schedule (i.p. 0.5 mg/kg/day for 7 days), we also found that treatment of 6-month-old C9BAC mice with JQ1 was well tolerated and did not led to any apparent toxicity or aberrant phenotypical alterations, including changes of total exploration time in the OLM test (Fig. 5e). Strikingly, all C9BAC mice showed significant improvements in the OLM task after 7 days of JQ1 treatment (Fig. 5b–d). This was especially evident when we displayed the increased normalized exploration time towards the displaced object for each individual animal before and after JQ1 treatment (Fig. 5c). Together, these results demonstrate that treatment with BETi JQ1can revert the hippocampal-dependent spatial memory impairment of C9BAC mice.

## Discussion

**Fig. 6:**
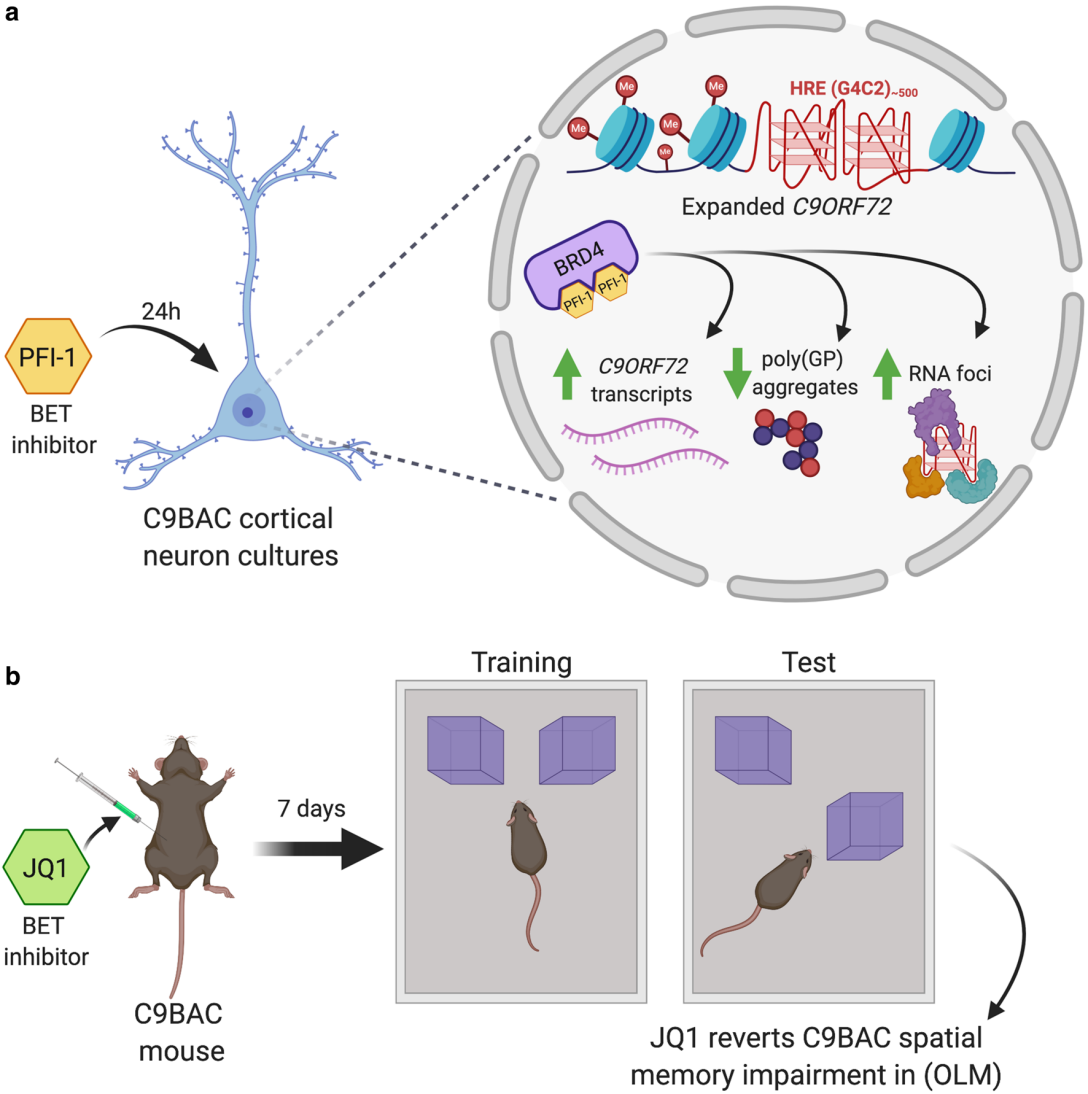
Proposed model describing the effects of BETi PFI-1 and JQ1 in C9BAC cultures and mice. PFI-1 and JQ1 are highly potent pan-BETi and antagonize BET family members BRD4 > BRD2/3 > BRDT. **a** Treatment of primary cortical cultures from C9BAC mice with PFI-1 (5 μM for 24 h) enhances the expression of the human *C9ORF72* gene in neurons, leading to increases in *C9ORF72* transcripts, reduction in poly(GP) aggregates, and accumulation of RNA foci. The methylation of the human *C9ORF72* gene promoter region represents an enrichment of several repressive histone modifications, including H3K9me3 and H3K27me3 in the histone tails of the depicted nucleosomes. Higher-order DNA structures, called G-quadruplexes, found in the G4C2 hexanucleotide repeat expansions (HRE) of the *C9ORF72* gene body are also shown. For simplicity only BET family member BRD4 is shown and the ET domain omitted. **b** Treatment of 6-month-old C9BAC mice with JQ1 (i.p. 0.5 mg/kg/day for 7 days) rescues hippocampal-dependent cognitive spatial memory, as measured by the object location memory (OLM) task

Using C9ALS/FTD luciferase reporter cell lines generated by our team and comprising the human *C9ORF72* gene with pathological G4C2 repeat expansions, we assessed whether a specific set of small molecules targeting and inhibiting chromatin-regulating proteins can modulate *C9ORF72* gene expression. Of the 14 different epidrugs tested, bromodomains inhibitors consistently increased *C9ORF72* transcription in the C9ALS/FTD luciferase reporter cell lines. Using primary cortical cultures from C9BAC mice, we further showed that treatment with the BETi PFI-1 results in (1) increased expression of 3 human mutant *C9ORF72* transcript variants (V1-V3), (2) reduced presence of poly(GP)-DPR inclusions, and (3) increased accumulation of intranuclear RNA foci. Studies in vivo further demonstrated that administration of the BETi JQ1 rescued hippocampal-dependent cognitive impairments in C9BAC mice. The data is summarized in a model (Fig. 6). Our findings place BETi as a potential therapy for C9ALS/FTD by ameliorating *C9ORF72*-associated pathological and behavioral abnormalities.

Our data indicate that BETi PFI-1 treatment rescues both haploinsufficiency and DPR-mediated toxicity in C9BAC neurons. Currently, there are two principal explanations for how PFI-1 treatment could reduce DPRs through increasing *C9ORF72* gene expression. First, PFI-1-mediated increases in *C9ORF72* mRNA and hence protein levels can maintain the autophagy capacity sufficiently high to degrade poly(GP)-DPRs in C9BAC neurons. This mechanism is supported by recent studies demonstrating that reduced C9ORF72 protein levels lead to autophagy deficits and consequently impaired clearance of DPRs [14, 54]. Second, PFI-1-mediated increases in intronic repeat transcripts may consequently lead to more RNA foci that, in turn, prevent production of toxic DPRs in the cytoplasm. RNA foci have been mainly classified as neuropathological hallmarks, as they are proposed to sequester RNA-binding proteins and splicing factors, including RanGap1, ADARB2, nucleolin, hnRNP-H, hnRNPA1, hnRNPA3, ASF/SF2, Pur-α, Zfp106, SRSF2, ALYREF and hnRNP H1/F [11]. However, several studies in humans and flies indicate that nuclear RNA foci by themselves are not toxic. Thus, a correlation study in a large cohort of *C9ORF72* expansion carriers found that the abundance of antisense RNA foci in the frontal cortex was associated with a delayed age of disease onset [24]. And intriguingly, two genetic studies using sense transcript (antisense was not tested) in *Drosophila* distinguished the toxicity from repeat RNA as compared to the DPR protein [26, 27]. These studies reported that while polyA^+^ transcripts encoding DPRs cause robust neurodegeneration, neuronal survival was intact when cells expressed repeat transcripts that could not produce DPRs [26]. Another study expressing expanded G_4_C_2_ repeats in an intron resulted in the accumulation of numerous nuclear RNA foci but with no or little global effects on RNA processing and cellular toxicity [27], suggesting that, at least in *Drosophila*, nuclear RNA foci sequesters repeat transcripts, hence preventing their transport to the cytoplasm and the subsequent production of toxic DPRs. Based on these findings, we propose that the increased intranuclear RNA foci in PFI-1 treated C9BAC cortical neurons could function as a neuroprotective sponge for pathological *C9ORF72* repeat transcripts and hence impede the generation of toxic poly(GP)-DPR inclusions. It is currently not known if both sense and antisense repeat transcripts could give rise to beneficial effects. In the case of C9BAC neurons, poly(GP)-DPRs are abundant and are produced from the sense (G4C2) and antisense (G2C4) transcripts.

All 6 bromodomain inhibitors evaluated in our epidrug screen significantly increased *C9ORF72* transcription in the pathological G4C2 repeat expansions (90 and/or 200 HRE). Bromodomain-containing proteins belong to an evolutionarily conserved family encompassing approximately 61 types of domains in humans; these domains are present in 46 different proteins, some of them containing up to 6 bromodomains [66, 67]. In general, bromodomains are divided into 8 sub-families (I-VIII) based on sequence and structural conservation [60, 68, 69]. One of these families, bromodomain family II, or also named the BET bromodomain family, shares a common domain architecture comprising a pair of N-terminal bromodomains (BDs: BD1 and BD2) and an extra-terminal (ET) domain [67]. Importantly, five of the six bromodomain inhibitors—PFI-1, JQ1, RVX-208, I-BET151 and OTX015—that were found here to increase *C9ORF72* gene expression are selective and potent inhibitors of this BET bromodomain family [59, 60]. Our results extend a previous study by Zeier et al. [42], that initially proposed BET and other bromodomain inhibitors as regulators of the *C9ORF72* locus in C9ALS. That study, which was focused on the effect of JQ1 and I-BET151 (and did not include PFI-1), concluded that these compounds can substantially increase *C9ORF72* gene expression (8–tenfold) in fibroblasts derived from C9ALS patients. Treatment of iPSC-derived motoneurons from C9ALS patients with JQ1 and I-BET151 showed a rather modest effect on *C9ORF72* gene expression (1.3–3 fold) [42].

The BET family consists of four conserved mammalian members, namely, BRD2, BRD3, BRD4 and testis-specific BRDT. While PFI-1 and JQ1 are structurally different, both are highly potent pan-BETi and antagonize BRD4 > BRD2/3 > BRDT [60, 67]. BET proteins, acting through BD1 and BD2, recognize (or ‘read’) acetylated lysines (Kac) in the tails of the four histone proteins and in non-histone proteins, including transcription factors [66, 67, 70]. Furthermore, the ET domain of BET proteins mediates protein–protein interactions and recruits transcriptional and epigenetic regulatory complexes to the acetylated protein target, contributing to processes such as chromatin remodeling and transcription [66, 67]. BETi disrupt the BET-Kac interaction, leading to the release of BET proteins from the chromatin. In general, BETi result in global and specific transcriptional repression, particularly of oncogenes, and hence BETi have been used in over 20 clinical trials for cancer [67, 71]. Other studies, however, have reported that subsets of genes are upregulated by BETi [68, 72–75]. The mechanisms underlying this locus-specific up-regulation or de-repression of transcription by BETi are not well understood, in part because the comprehension of the BET protein complex formation at target genes is still limited [67]. Recently, the interactome of each BET protein (BRD2-4 and BRD-T) was established using proteomic analyses in cell lines (HEK293 cells and chronic myeloid leukemia K652 cells) in the presence or absence JQ1 [70]. In the absence of JQ1, 650 high-confidence BET interactors were identified and enriched interactomes generated for each BET protein, among them not only transcriptional activators, such as the Positive Transcription Elongation Factor b (P-TEFb), but also transcriptional repressors including the Negative Elongation Factor b (NELFb) [70]. Interestingly, NELFb has been shown recently to play a relevant role in constraining transcription as it mediates RNA polymerase II pausing at proximal gene promoter regions [76]. Hence, release of NELFb from these promoters is strongly associated with transcriptional enhancement.

JQ1 treatment induced extensive changes in the BET interactome, which now include novel Kac-independent interactions with components of the transcription initiation complex (PIC) like Mediator, TFIID, TFIIH, and RNAPII [70]. Future studies in C9ALS/FTD neurons will be needed to address whether BETi induce de-repression of the silent expanded *C9ORF72* gene by promoting the release of repressors, stimulating the interaction with specific activators, or both. Additionally, it is important to consider that BETi may be increasing the expression of the mutant *C9ORF72* gene indirectly, perhaps by altering the expression of upstream regulators of this gene. BETi may also restore transcriptional and epigenetic alterations observed in C9ALS/FTD patients and model systems, including widespread changes in mRNA expression [46, 77, 78] and global loss of the repressive H3K9me3 mark in several brain regions, including in the spinal cord, motor cortex and hippocampus [61].

Our studies in vivo demonstrate that BETi JQ1 improves the object location preference (OLM) in C9BAC mice, indicating that this epidrug can rescue, at least in part, hippocampal-dependent cognitive deficits in spatial learning and memory found in C9BAC mice. In contrast, a comparable JQ1 treatment in wild-type mice showed that this BETi can block novel object preference (NOR) [65], a task that studies non-spatial learning of object identity and relies on multiple brain regions, with some controversy on the involvement of the hippocampus [62]. Together, these data suggest that the role of BET proteins in non-spatial long-term memory formation is likely associated with a variety of brain regions whose precise roles in hippocampal function are not well understood. It will be interesting to evaluate if JQ1 treatment in C9BAC mice increases the expression of *C9ORF72* transcripts (testing both the human mutant *C9ORF72* gene and the mouse wild-type *C9orf72* gene), reduces poly(GP)-DPR inclusions and enhances intranuclear RNA foci. Similar studies with PFI-1 could be undertaken although such preclinical animal research should be accompanied by pharmacokinetic analysis of this epidrug in the brain, as shown for JQ1 [64].

While there is a risk that using JQ1 or equivalent BETi to treat systemic diseases (e.g. several types of cancer) may negatively affect cognition in normal subjects, our data nonetheless supports a potential use of JQ1-like epidrugs as a therapeutic strategy in C9ALS/FTD. In the future, it will be important to evaluate whether any additional behavioral abnormality found in C9ALS/FTD patients (mostly absent in mouse models), including hyperactivity, anxiety, antisocial behavior, and motor deficits, may be also ameriolated by BETi treatment.

## Methods

### Animals

All protocols involving mice were carried out according to NIH, ARRIVE and ANID/CONICYT guidelines and were approved by the Ethical and Bio-security Committees of Universidad Andrés Bello. C9BAC mice expressing *C9ORF72* genomic DNA sequence derived from a familial C9ALS/FTD patient under a B6SJLF/J background were used together with the non-transgenic littermates (Ntg) as controls [55]. Transgenes in the C9BAC mice were identified by PCR using the primers for Orf72e3: forward 5′ TTA ATT TCC TAC CCC TGC CC 3′; reverse 5′ AGG CCT TGA CAA ATG TAG CC 3′ and for Mm10chr3: forward 5′GCC TCA CCt CCT AAG AGC CTA 3′; reverse 5′ CCT TTG TGT CAC ACG GAT ATC 3′ [55].

### Generation of SH-SY5Y G4C2 cell lines

Three different stable SH-SY5Y cell lines were generated based a construct of the human *C9ORF72* gene that included 4 kb of the promoter region and exons 1a through 2 with 10, 90 or 200 hexanucleotide expansions. This was cloned into the PGL4.17[luc2/Neo] plasmid (Promega), in which expression of luciferase is driven by the *C9ORF72* promoter with the associated G4C2 repeat. Plasmids were transfected into SH-SY5Y cells and stable lines were selected using G418 (Gibco). These were designated SH-SY5Y WT, SH-SY5Y LE90 and SH-SY5Y LE200, respectively designating the G4C2 repeat motif sizes (10, 90 and 200).

### SH-SY5Y G4C2 cell line maintenance and epidrug treatment

Cells were cultured in DMEM-F12 (Gibco, 12,400–024) 10% FBS (HyClone, SH30071.03) and were selected by adding G418 to culture medium (HyClone, SV30068.02). When SH-SY5Y G4C2 cell lines reached 80% confluence they were treated for 24 h with 14 different epidrugs: details on the company, lot number and concentrations of each epidrug used to treat SH-SY5Y cells are shown in Additional file 1: Table S1. All epidrugs were freshly prepared and dissolved in DMSO (Sigma, D2650-5) and then diluted in sterile ultrapure water (Invitrogen, 10,977–023) before using them in cultures. The DMSO volume concentrations (v/v) used was dependent on the final epidrug concentration: 0.005% DMSO for 0.5 µM (only for GSK3430), 0.025% DMSO for 2.5 µM (only for OTX015), 0.25% DMSO for 25 µM (only for RXV208) and 0.05% DMSO for 5 µM (rest epidrugs). After the 24 h treatment, cells were harvested following the instructions of Dual-Luciferase® Reporter Assay System Kit (Promega, E1960). Relative light units were normalized to total protein levels and quantified with Pierce BCA Protein Assay kit (Thermo Fisher Scientific, 23255).

### Primary C9BAC cortical neuronal cultures

Prior to preparing the primary cultures (2.5 h before), a small tail tissue sample of each P0-2 mouse was performed to identify transgenic C9BAC mice from their non-transgenic littermates by PCR (see above for primers; for quick DNA purification see [79]. Cerebral cortical cultures were generated as previously described [80] with some modifications. Briefly, cortices from individual brains of C9BAC mice were placed into ice-cold PBS 0.01 M (Gibco, Cat. No. 14185–052) containing 50 μg/ml penicillin/streptomycin (Gibco, Cat. No. 15070–063) and dissected into small pieces. Cortical tissue (one cortex for one 24-well plate) was digested in 2 ml PBS 0.01 M containing 50U of papain (Worthington, Cat. No. LS03126), 17 μg/μl L-Cysteine (Sigma, Cat. No. C7755) and 25 μl/ml DNAase (Roche, Cat. No. 1010415900) at 37 °C for 15 min. Cells were transferred to a 15 ml tube containing Neurobasal Medium (Gibco 21103-049), 1% penicillin–streptomycin (Gibco, Cat. No. 15070-063) and 2-Amino-5-phosphonopentanoic acid (5 mM; Sigma, Cat. No. A5284), re-suspended by mechanical agitation through fire-polished glass Pasteur pipettes of different tip diameters, and counted: 250,000 cells/well were plated on poly-L-lysine-coated 24-well plates (1 mg/ml; 30,000–70,000 MW; Sigma, Cat. No. P2636). Each well contained Neurobasal Medium (Gibco, 21103-049) supplemented with B27 (Gibco, 17504-044) and 0.5 × GlutaMax (Gibco, 3505006) in the presence of 100 μg/ml Primocin (InvivoGen, Cat. No. ANT-PM-1). One day after plating, cells were treated during 24 h for once with 20 μM cytosine arabinoside in order to prevent microglial proliferation. After that, all media was changed. Half of the media was replaced every 2–3 days. Treatments with PFI-1 (5 μM) were performed at 9 DIV for 24 h.

### TaqMan qRT-PCR

Total RNA was extracted from 10 DIV primary C9BAC and non-transgenic cortical cells (2 million/condition) using Trizol (Thermo Fisher Scientific, 15596018) following manufacturer instructions. Reverse transcription to cDNA was performed with High-Capacity RNA-to-cDNA™ Kit (Thermo Fisher Scientific, 4387406). Quantitative PCR was performed using TaqMan Fast Advanced Master Mix following manufacturer instructions. Every transcript variant of the human *C9ORF72* gene were measured with a specific probe for each variant (V1, V2 and V3) and one probe that hybridizes with all variants (Vall) (Thermo Fisher Scientific). As an endogenous control we used a mouse *Hprt* commercial assay (Thermo Fisher Scientific, Mm01545399_m1). The obtained results were processed by using the delta delta Ct method. Details about the different probes are depicted in Additional file 1: Table S2.

### Poly(GP) immunostaining

Immunostaining to detect poly(GP) dipeptides on C9BAC cortical neurons as previously described [18]. Briefly, primary C9BAC and non-transgenic cortical cells (10 DIV) were fixed with PFA 4% and sucrose 4%, and then permeabilized with Triton X-100 (Sigma, T8787) 0.1% v/v for 20 min. Next, cells were blocked with Serum-Free Protein Blocking solution (DAKO, X0909) for 30 min before primary antibody incubation. Poly(GP) was detected with anti-poly(GP) polyclonal antibody (1:20.000, Merck, ABN455) and neurons were labeled with anti-microtubule associated protein 2 monoclonal antibody (MAP2 1:200; Santa Cruz, sc-74421); primary antibodies were incubated overnight at 4ºC. Secondary antibody incubations were performed for 2 h using Alexa 488 (1:500, Company, A11034) to detect poly(GP) and Alexa 546 for MAP2 (1:500, Company, A11003). Coverslips were mounted using Prolong® Gold antifade reagent with DAPI (Thermo Fisher Scientific, P36931).

Images of primary cortical neuron cultures were taken on an Olympus FV1000 confocal microscope with a 60 × oil objective (NA = 1.35; UPLS APO) and a z-step of 0.5 μm optical sections (velocity scan 12.5 μs/per pixel; resolution 1024 × 1024 pixels, equivalent to 212 μm × 212 μm). The following laser wavelengths were used to detect DAPI (Ex 405 nm and Em 422–475 nm), MAP2-Alexa546 (Ex 543 nm and Em BA560–660 nm) and poly(GP)-Alexa488 (Ex 488 nm and Em 500–530 nm). Maximum intensity projections of confocal z-stack images of whole nuclei (containing 8–10 stacks) were analyzed. To validate the specificity of the anti-poly(GP) antibody, customized synthesis of purified poly(GP)_8_ dipeptide was obtained from Abmgood. For the pre-absorption assay, the anti-poly(GP) antibody was pre-incubated (30 min at room temperature) with an excess of the poly(GP)_8_ peptide in Serum-Free Protein Blocking solution (DAKO, X0909) before performing the immunostaining assays as described above. Fiji ImageJ software (8 bits, measuring intensity from 0 to 150) was used to measure the poly(GP) nuclear fluorescence intensity. Line scans were drawn in ImageJ to quantify the relative intensities of the fluorescence signal for poly(GP). Maximum intensity projections of confocal z-stack images of whole nuclei were analyzed. At least 30 neuronal nuclei were analyzed for each condition.

### RNA fluorescence in situ hybridization (FISH)

RNA FISH with a probe targeting *C9ORF72* sense transcript on primary C9BAC neurons was performed as previously described [55]. Briefly, primary C9BAC and non-transgenic cortical cells (10 DIV) were fixed with PFA 4% and then permeabilized with Triton X-100 (Sigma, T8787) 0,1% v/v for 20 min. Next, cells were incubated for 1 h with pre-hybridization buffer followed by overnight hybridization at 55ºC with a DNA probe targeting *C9ORF72* sense transcript coupled with a Cy3 5′ end tag ((/5Cy3/-GGCCCC)4, IDT 162,193,262). Coverslips were mounted using Prolong® Gold antifade reagent with DAPI (Thermo Fisher Scientific, P36931). Images of primary cortical neuron cultures were taken on a Leica TCS SP8 confocal microscope with a 63 × oil objective and × 4 × digital zoom (NA = 1.4; HC PL APO CS2) and a z-step of 0.5 μm optical sections (velocity scan 600 Hz; resolution 1024 × 1024 pixels, equivalent to 46.18 μm × 46.18 μm). The following laser wavelengths were used to detect DAPI (Ex: 405 nm and Em: 410–483 nm), RNA-foci were detected by Cy3 dye (TRITC, Ex: 532 nm and Em: 580–625 nm). Brightfield images were taken to identify cell body. Maximum intensity projections of confocal z-stack images of whole nuclei (containing 10 stacks) were analyzed. Fiji ImageJ software (8 bits, measuring foci from 0 to 50) was used to quantify the number of RNA foci. The quantification of foci (≥ 0.20 μm) was carried out under threshold conditions using the analyze particle plugin. Maximum intensity projections of confocal z-stack images of whole nuclei were analyzed. At least a total of 30 neuronal nuclei were analyzed for each condition.

### Object location memory (OLM) tests

The single trail OLM test was performed as previously described [61, 63]. Briefly, this test consists of three steps: habituation, a training session (10 min) and finally a test session (10 min) 24 h later. Mice were individually habituated in an apparatus that contained a cage inside of a soundproof chamber. In the training session, the cage contained two identical objects, termed “non-displaced objects” (ND). During the test session, one object was changed its place in the chamber, termed “displaced object” (D). The exploration time was recorded and defined as the time that the animal spent sniffing or touching the object with the nose and/or forepaws. The ‘discrimination index’ was calculated by the time spent in exploring the displaced object (D) over the total time exploring both objects (D + ND).

### JQ1 injections

A group of 6-month-old male mice C9BAC mice were first subjected to the OLM test to show impaired spatial memory. Then, the same group of mice were injected intraperitoneally with (+)-JQ1 (Sigma-Aldrich, SML1524), as described previously [64]. Briefly, JQ1 was dissolved in DMSO at 5 mg/ml and then diluted 1:10 in 10% (2-Hydroxypropyl)-b-cyclodextrin (Sigma-Aldrich, H107). The mixture was injected i.p. into mice at a final concentration of 0.5 mg/kg daily for 7 days in a row. Animals were weighed daily before injections (ranging from 28 to 35 g) and fed ad libitum.

### Statistical analyses

Statistical analyses were performed using GraphPad Prism 5 software. One-way ANOVA followed by the Bonferroni post-hoc was utilized when making multiple (three or more) comparisons. Unpaired Student’s *t*-test was performed when two populations were examined. In all figures, data is reported as mean ± S.E.M.; *P < 0.05, **P < 0.01 and ***P < 0.001 compared to controls, either wild-type or Ntg cells or DMSO-treated (as indicated). In some experiments (Fig. 1c, d), ^#^*P* < 0.05, ^##^*P* < 0.01 and ^###^*P* < 0.001 are shown to compare treated to untreated SH-SY5Y LE90/200 cells (as indicated in legends).

## Supplementary Information

**Additional file 1: Fig. S1**. Bromodomain inhibitors enhance C9ORFF72 gene promoter-driven expression in SH-SY5Y LE200 G4C2 reporter cell lines. **Table S1**. Epidrugs used in screening. **Table S2**. TaqMan probes information.

## Data Availability

All data generated or analyzed during this study are included in this published article.

## Abbreviations

A: Alanine
ALS: Amyotrophic Lateral Sclerosis
BAC: Bacterial Artificial Chromosome
BET: Bromodomain and Extra-Terminal motif
BETi: BET inhibitor(s)
C9BAC: C9ALS/FTD BAC mouse model
D: Displaced
DIV: Days in vitro
DMSO: Dimethyl Sulfoxide
DPR: Dipeptide-repeat
G2C4: GGCCCC
G4C2: GGGGCC
G: Glycine
FBS: Fetal bovine serum
FTD: Frontotemporal dementia
G2C4: GGCCCC
G4C2: GGGGCC
G: Glycine
HRE: Hexanucleotide repeat expansion
H3K9me3: Histone 3 lysine 9 trimethylation
H3K27me3: Histone 3 lysine 27 trimethylation
i.p.: Intra peritoneal
MAP2: Microtubule Associated Protein 2
NeuN: Neuronal nuclei
ND: Non-displaced
Ntg: Non-transgenic
OLM: Object Location Memory
P: Proline
PFA: Paraformaldehyde
Poly(GA): poly(Glycine-Alanine)
Poly(GP): poly(Glycine-Proline)
Poly(GR): poly(Glycine-Arginine)
Poly(PA): poly(Proline-Alanine)
Poly(PR): poly(Proline-Arginine)
R: Arginine
RAN: Repeat-associated non-AUG-dependent
iPSC: Induced pluripotent stem cell
