# Supplementary material for: BET bromodomain inhibitors PFI-1 and JQ1 are identified in an epigenetic compound screen to enhance C9ORF72 gene expression and shown to ameliorate C9ORF72-associated pathological and behavioral abnormalities in a C9ALS/FTD model
Source: Clin Epigenetics. 2021 Mar 16;13:56. doi: 10.1186/s13148-021-01039-z (PMC7962347; doi:10.1186/s13148-021-01039-z)
Supplement: Supplementary file 1 — Additional file 1: Fig. S1. Bromodomain inhibitors enhance C9ORFF72 gene promoter-driven expression in SH-SY5Y LE200 G4C2 reporter cell lines. Table S1. Epidrugs used in screening. Table S2. TaqMan probes information. [file 13148_2021_1039_MOESM1_ESM.pdf]

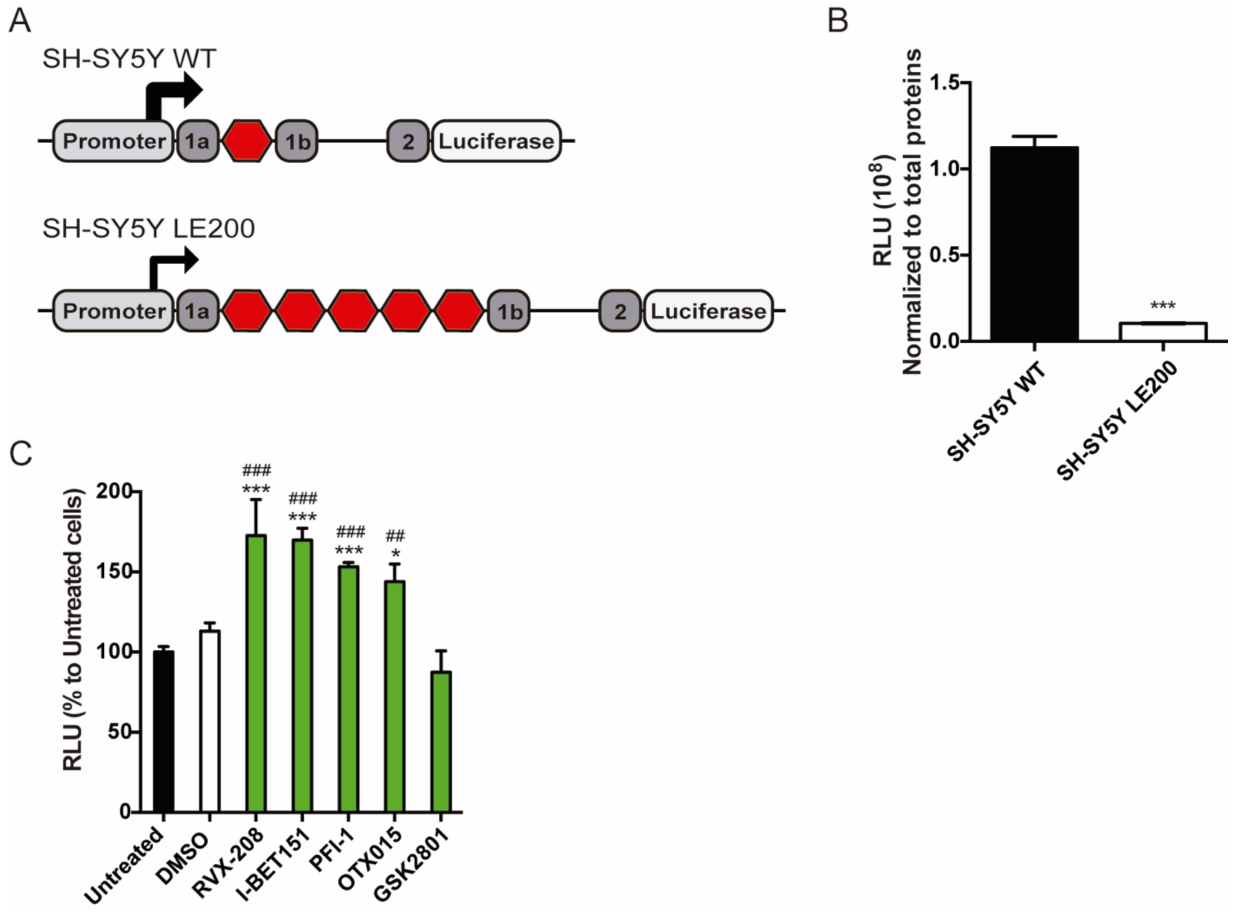

Supplementary Figure 1: Bromodomain inhibitors enhance *C9ORFF72* gene promoter-driven expression in SH-SY5Y LE200 G4C2 reporter cell lines.

(A) Schematic representation of two SH-SY5Y cell lines stably expressing a segment of the human *C9ORF72* gene, including the promoter region and exons 1a through 2, driving the expression of a luciferase reporter gene. Between exons 1a and 1b, increasing G4C2 hexanucleotide repeat expansions (red hexagons) were inserted: 8 (SH-SY5Y WT) or 200 (SH-SY5Y LE200). (**B**) Quantification of the basal luciferase activity in each cell line. Values were normalized to total protein concentration and displayed as relative luciferase unit (RLU). Bars represent mean±S.E.M. ****P*<0.001 relative to SH-SY5Y WT cells, unpaired Student's *t*-test (n=3 independent experiments). (**C**) Quantification of luciferase activity at the SH-SY5Y LE200 cells following an incubation for 24 h with 5 selective small molecule inhibitors targeting bromodomains (green bars). As a control, SH-SY5Y LE200 cells were also treated with 0.05% (v/v) DMSO (white bar), the same concentration used as the vehicle for most of the epidrugs. Luciferase activity in the treated SH-SY5Y LE200 cells was normalized to untreated SH-SY5Y LE200 cells (black bar). Bars represent mean±S.E.M. **P*<0.05 and ****P*<0.001 relative to DMSO, or ##*P*<0.01 and ###*P*<0.001 relative to Untreated, one-way ANOVA (n=3 independent experiments).

**Table 1. Epidrugs used in screening**							
Epidrug	Concentration	Company/ Cat #	Reference	Target protein	IC50	logP	Molecular structure and weight (g/mol)
PFI-1	5µM	BioVision 2203-1	Picaud et al., *Cancer Res.*, 2013	BRD4(BD1)	220nM	1.3	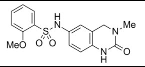 347.1
(+)-JQ1	5µM	Tocris 4499	Filippakopoulos et al., *Nature*, 2010	BRD4(BD1)	77nM	4.0	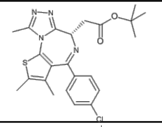 456.1
RVX-208	25µM	MedChem HY-16652	Picaud et al., *Proc Natl Acad Sci*, 2013	BRD4(BD2)	510nM ± 41nM	2.3	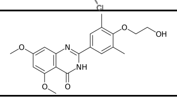 370.4
GSK2801	5µM	Sigma SML0768	Chen et al., *J. Med. Chem.*, 2016	BAZ2A, BAZ2B	260nM 140nM	3.5	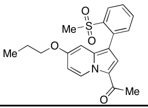 371.5
I-BET151	5µM	BioVision 2220-1	Dawson et al., *Nature*, 2011	BRD2 BRD3 BRD4	500nM 250nM 790nM	2.7	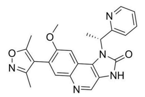 415.4
OTX015	2,5µM	Sigma SML1605	Noel et al., *Mol. Cancer Ther.*, 2013	BRD2 BRD3 BRD4	99–112nM	4.5	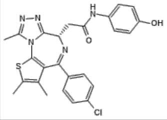 491.1
GSK343	0,5µM	BioVision 2281-1	Verma et al., *ACS Med. Chem. Lett.*, 2012	EZH2	4nM	3.23	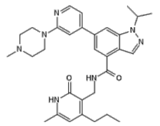 541.3
GSK126	5µM	BioVision 2282-1	McCabe et al., *Nature*, 2012	EZH2	9.9nM	3.9	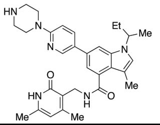 526.7
UNC1999	5µM	BioVision 2449-5	Xu et al., *Blood*, 2015	EZH2	2nM	4.01	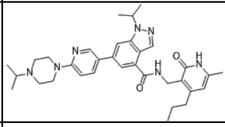 569.7
EPZ-6438	5µM	BioVision 2383-5	Knutson et al., *Proc Natl Acad Sci*, 2013	EZH2	11nM	4.2	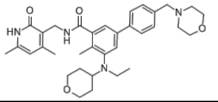 572.7
UNC669	5µM	BioVision 2620-5	Herold et al., *J. Med. Chem.*, 2011	L3MBTL1	6µM	-	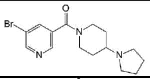 338.2
UNC1215	5µM	BioVision 2252-1	James et al., *Nat. Chem. Biol.*, 2013	L3MBTL3	40nM	4.17	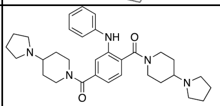 529.3
TCP	5µM	R&D 3852/10	Lee et al., *Chem. Biol.*, 2006	LSD1	200nM	1.5	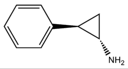 133.1
C646	5µM	BioVision 1948-1	Bowers et al., *Chem. Biol.*, 2010	P300/CBP	1µM	4.2	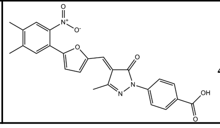 445.4

Supplementary Table 2: TaqMan probes information.

TaqMan probe	Type of assay	Sequence	Primers	Assay code
V1	Commercial assay	AGATGACGCTT GATATCTCCGG AGC	Not shown	Hs00331877_m1, Thermo Fisher Scientific
V2	Custom assay	CGACTCTTTGC CCACCG	F:AGGCGGTGGCG AGTGGATA R:TTGGAGCCCAA ATGTGCCTTA	Thermo Fisher Scientific
V3	Custom assay	CCACCGCCATC TC	F:GCGGGGTCTAG CAAGAGCAG R:TTGGAGCCCAA ATGTGCCTTA	Thermo Fisher Scientific
Vall	Commercial assay	AGAATATGGAT GCATAAGGAA AGAC	Not shown	Hs00376619_m1
HPRT	Commercial assay	Not shown	Not shown	Mm01545399_m1
